# Targeting C12ORF49‐Mediated Ferroptosis in Hepatocellular Carcinoma

**DOI:** 10.1002/jgh3.70353

**Published:** 2026-02-09

**Authors:** Yuexin Liu, Lizhou Jia, Liu Yang, Zhang Ning, Yanmei Li

**Affiliations:** ^1^ Department of Gastroenterology Affiliated Hospital of Inner Mongolia Medical University Hohhot City Inner Mongolia Autonomous Region China; ^2^ Central Laboratory, Bayannur City Hospital Bayannur City Inner Mongolia Autonomous Region China

**Keywords:** C12ORF49, ferroptosis, hepatocellular carcinoma (HCC), lipid metabolism, SCD1, sorafenib, SREBP1

## Abstract

Hepatocellular carcinoma (HCC) remains a leading cause of cancer‐related mortality worldwide, underscoring the urgent need for novel therapeutic strategies. Recent studies have highlighted the pivotal role of ferroptosis, an iron‐dependent form of regulated cell death driven by lipid peroxidation, in cancer biology. C12ORF49, an emerging regulator of lipid metabolism, has gained attention for its influence on HCC cell survival and tumor progression. Specifically, C12ORF49 modulates the SREBP1/SCD1‐mediated fatty acid metabolic pathway, which in turn suppresses ferroptosis, facilitating tumor cell survival and resistance to conventional therapies. Despite advances in understanding ferroptosis pathways, the complex interplay between lipid metabolism regulators like C12ORF49 and ferroptotic signaling in HCC remains incompletely understood. This review comprehensively summarizes current knowledge on the molecular mechanisms by which C12ORF49 intersects with ferroptosis signaling, highlighting its impact on lipid metabolic reprogramming in HCC. Furthermore, we explore the potential of targeting C12ORF49 to enhance the efficacy of existing treatments such as Sorafenib, a frontline systemic therapy for advanced HCC. By elucidating the crosstalk between C12ORF49 and ferroptosis pathways, this article aims to provide a theoretical framework and identify promising therapeutic targets for precision medicine approaches in hepatocellular carcinoma.

## 1 Introduction

Ferroptosis, a regulated cell death modality driven by iron‐dependent lipid peroxidation, has emerged as a critical vulnerability in cancer biology [1, 2]. Unlike apoptosis, ferroptosis execution is strictly dependent on the oxidation of polyunsaturated fatty acid (PUFA)‐containing phospholipids in cellular membranes [3, 4]. While tumor cells often exhibit susceptibility to ferroptosis due to altered iron metabolism and oxidative stress [5, 6], they can also hijack metabolic pathways to modulate the tumor microenvironment (TME) and immune responses, thereby influencing progression and metastasis [7, 8, 9]. However, the efficacy of ferroptosis‐inducing therapies is intrinsically linked to the tumor's lipid metabolic state [10, 11, 12]. Crucially, the cellular balance between saturated fatty acids (SFAs), monounsaturated fatty acids (MUFAs), and PUFAs acts as a rheostat for cell death: PUFAs serve as the primary substrates for lethal peroxidation, whereas MUFAs act as competitive inhibitors that suppress ferroptosis [13, 14, 15, 16, 17, 18]. Therefore, enzymes governing fatty acid desaturation, such as Stearoyl‐CoA Desaturase 1 (SCD1), are pivotal determinants of ferroptosis sensitivity [19, 20], and their dysregulation contributes significantly to plasticity and drug resistance [21, 22].

At the nexus of these metabolic and cell death pathways lies C12ORF49 (also known as SPRING or POST1), a recently identified master regulator of lipid homeostasis [23, 24, 25]. Functioning as an essential cofactor for Site‐1 Protease (S1P), C12ORF49 orchestrates the maturation of Sterol Regulatory Element‐Binding Proteins (SREBPs), the transcription factors that drive lipogenesis [23, 26, 27]. In Hepatocellular Carcinoma (HCC), C12ORF49 is upregulated and correlates with poor prognosis [28]. Mechanistically, it promotes the SREBP1/SCD1 axis, leading to the accumulation of MUFAs that shield cells from lipid peroxidation, thereby conferring resistance to ferroptosis inducers like Sorafenib [28, 29, 30]. This review aims to systematically elucidate the molecular crosstalk between C12ORF49 and ferroptosis pathways, highlighting its potential as a therapeutic target to re‐sensitize refractory HCC to treatment.

## 2 Biological Functions of C12ORF49 and Its Role in Lipid Metabolism

### 2.1 C12ORF49 Molecular Structure and Expression Characteristics

**FIGURE 1:**
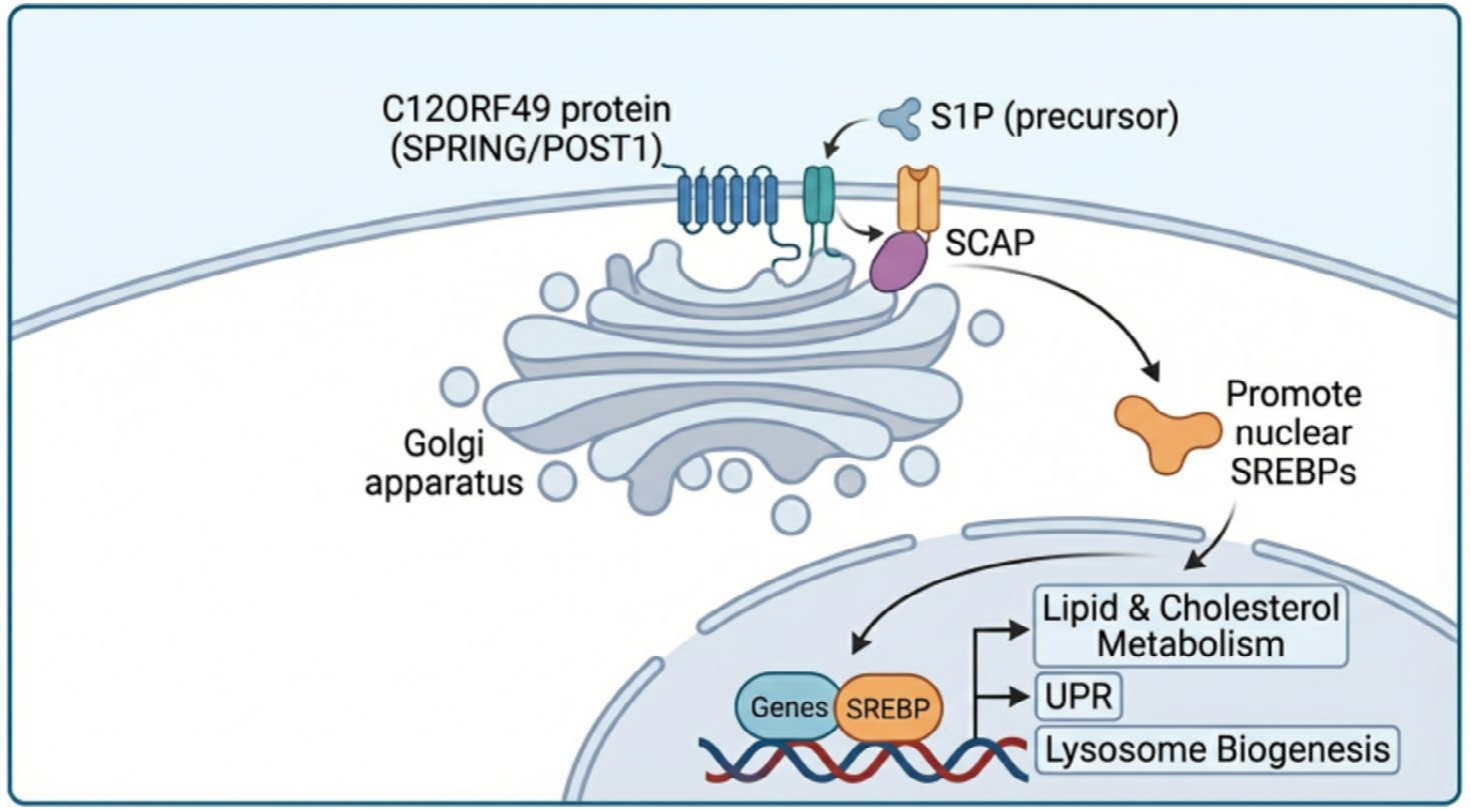
Schematic representation of the molecular function of C12ORF49 in regulating SREBP signaling. C12ORF49 is a Golgi‐resident membrane protein that acts as a critical cofactor. At the Golgi apparatus, C12ORF49 interacts with the S1P precursor and SCAP. This interaction facilitates the proteolytic processing and subsequent release of the mature, active nuclear form of SREBPs. The nuclear SREBPs then translocate into the nucleus and bind to target gene promoters to drive the transcriptional expression of genes involved in lipid and cholesterol metabolism, the unfolded protein response (UPR), and lysosome biogenesis.

C12ORF49 is a Golgi‐resident transmembrane protein that functions as a critical cofactor directly regulating the maturation of Site‐1 Protease (S1P). S1P is an indispensable enzyme for the activation of SREBP signaling, with SREBPs serving as master transcriptional regulators of cellular lipid synthesis and homeostasis (Figure 1) [23]. As illustrated, C12ORF49 facilitates the proteolytic maturation of S1P and the subsequent nuclear translocation of SREBPs, which then orchestrate the transcription of genes involved in lipid metabolism, the unfolded protein response (UPR), and lysosome biogenesis. Accumulating evidence indicates that C12ORF49 is aberrantly overexpressed in multiple malignancies, including colorectal cancer and breast cancer, where it correlates with tumor progression and poor prognosis [31, 32]. In HCC, C12ORF49 drives a lipogenic program that modulates cellular lipid composition, thereby shaping cellular sensitivity to ferroptosis [25, 33].

### 2.2 C12ORF49 Regulation of the SREBP Family Mechanisms

**FIGURE 2:**
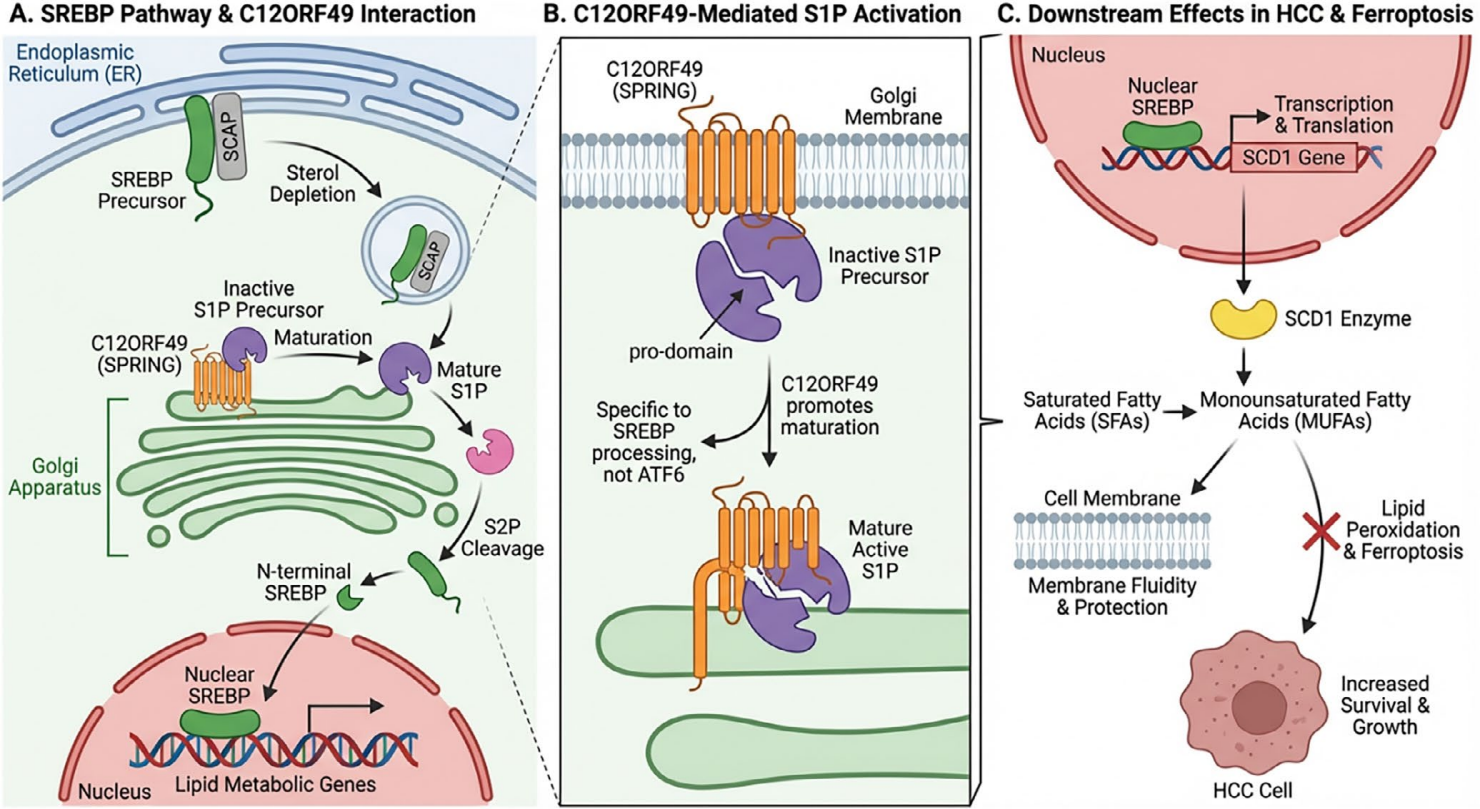
The regulatory role of C12ORF49 (SPRING) in SREBP pathway activation and downstream ferroptosis defense in HCC. (A) Interaction between the SREBP pathway and C12ORF49. Upon cellular sterol depletion, the SREBP precursor‐SCAP complex translocates from the endoplasmic reticulum (ER) to the Golgi apparatus. At the Golgi membrane, C12ORF49 interacts with the inactive S1P precursor, promoting S1P maturation. Mature S1P and subsequently S2P sequentially cleave SREBP, releasing the active N‐terminal nuclear SREBP fragment. This active fragment translocates into the nucleus to initiate the transcription of lipid metabolism‐related genes. (B) Detailed mechanism of C12ORF49‐mediated S1P activation. C12ORF49 localizes to the Golgi membrane and binds to the inactive S1P precursor, facilitating the removal of its inhibitory pro‐domain to generate the active mature S1P. As noted in the figure, this C12ORF49‐promoted maturation is specific for SREBP processing and does not affect ATF6. (C) Downstream effects in HCC and ferroptosis. Nuclear SREBP in the nucleus promotes the transcription and translation of the SCD1 gene. The resulting SCD1 enzyme catalyzes the conversion of SFAs into MUFAs. Increased levels of MUFAs enhance cell membrane fluidity and protection, thereby inhibiting lipid peroxidation‐driven ferroptosis. This metabolic reprogramming ultimately leads to increased survival and promoted growth of HCC cells.

SREBPs regulate cholesterol and fatty acid biosynthesis to maintain lipid homeostasis. They are synthesized as inactive precursors in the ER and translocate to the Golgi upon sterol depletion, where they undergo sequential cleavage by S1P and Site‐2 Protease (S2P). This cleavage releases the active nuclear form to drive lipogenic gene transcription (Figure 2) [23]. C12ORF49 (SPRING) is critical for this process; it interacts directly with the inactive S1P precursor at the Golgi membrane, facilitating its maturation into the active protease form. This C12ORF49‐mediated activation is specific to SREBP processing, as it does not affect other S1P substrates like ATF6 [23, 25, 26, 27]. Beyond maturation, C12ORF49 modulates downstream lipid enzymes, notably SCD1. SCD1 catalyzes the synthesis of MUFAs from SFAs. In HCC, increased C12ORF49 expression enhances SREBP1 activity and upregulates SCD1, promoting MUFA production. This metabolic reprogramming inhibits ferroptosis by reducing membrane susceptibility to lipid peroxidation, thereby supporting tumor survival. Functional studies confirm that C12ORF49 knockdown impairs this axis, sensitizing cells to ferroptosis and impairing tumor progression [28].

### 2.3 C12ORF49‐Mediated Lipid Metabolism Reprogramming

**FIGURE 3:**
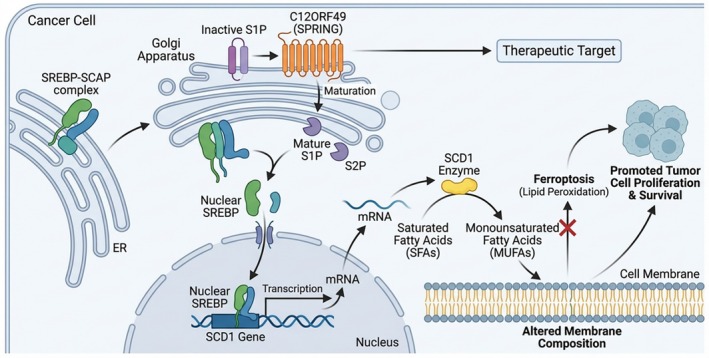
C12ORF49‐mediated lipid metabolism reprogramming. (A) Upstream Regulation: Within the Golgi apparatus, the C12ORF49 protein (orange transmembrane protein) promotes the maturation of the inactive S1P precursor into its active Mature S1P form. (B) SREBP Activation: The SREBP‐SCAP complex translocates from the ER to the Golgi. Here, mature S1P, along with S2P, sequentially cleave the SREBP protein. (C) Transcriptional Control: The cleaved, active Nuclear SREBP fragment translocates into the nucleus, where it acts as a transcription factor, binding to the promoter of the SCD1 gene and driving its transcription into mRNA. (D) SCD1 Expression and Function: The SCD1 mRNA is translated into the SCD1 enzyme in the cytoplasm. The SCD1 enzyme catalyzes the desaturation of SFAs into MUFAs. (E) Membrane Remodeling and Ferroptosis Inhibition: The newly synthesized MUFAs are incorporated into the cell membrane, leading to an altered membrane composition. This altered composition, characterized by a higher MUFA content, is less susceptible to lipid peroxidation, thereby inhibiting ferroptosis (a form of regulated cell death). (F) Tumorigenic Outcome: The inhibition of ferroptosis and the presence of MUFAs support cancer cell survival and proliferation, ultimately promoting tumor growth. (G) Therapeutic Potential: Given its central role in this pathway, C12ORF49 is highlighted as a potential therapeutic target for cancer treatment.

Lipid metabolic reprogramming is a crucial adaptive mechanism in cancer. C12ORF49 serves as a central node in this process by modulating the SREBP1/SCD1 axis to regulate MUFA synthesis (Figure 3). MUFAs are essential for membrane fluidity and signaling, and their accumulation displaces PUFAs in membrane phospholipids. Since PUFAs are the primary substrates for ferroptotic lipid peroxidation, this C12ORF49‐driven shift toward a MUFA‐rich profile effectively shields HCC cells from ferroptosis [23, 28]. This reprogramming supports rapid membrane biogenesis for proliferation and provides survival signals under metabolic stress. Consequently, C12ORF49 depletion leads to defects in lipid homeostasis and tumor growth suppression, highlighting its potential as a therapeutic target to disrupt metabolic adaptations in liver cancer [24, 25, 26].

## 3 Ferroptosis Basics and Regulatory Mechanisms

### 3.1 Definition and Biological Characteristics of Ferroptosis

Ferroptosis is a distinct modality of regulated cell death defined by iron‐dependent phospholipid peroxidation, fundamentally differentiating it from apoptosis, necrosis, and autophagy. Cytologically, it is characterized by specific mitochondrial alterations, including shrinkage, increased membrane density, and vanishing cristae [34, 35, 36]. At the molecular level, the hallmark of ferroptosis is the catastrophic failure of antioxidant defenses—primarily the System Xc^−^/Glutathione (GSH)/Glutathione Peroxidase 4 (GPX4) axis. GPX4 is essential for reducing toxic lipid hydroperoxides; its inactivation leads to the overwhelming accumulation of reactive oxygen species (ROS) and lethal oxidative damage to cellular membranes [37, 38]. The core execution mechanism hinges on the dysregulation of iron homeostasis, leading to an iron‐dependent catalytic surge in lipid peroxidation [39].

### 3.2 Relationship Between Lipid Metabolism and Ferroptosis

**FIGURE 4:**
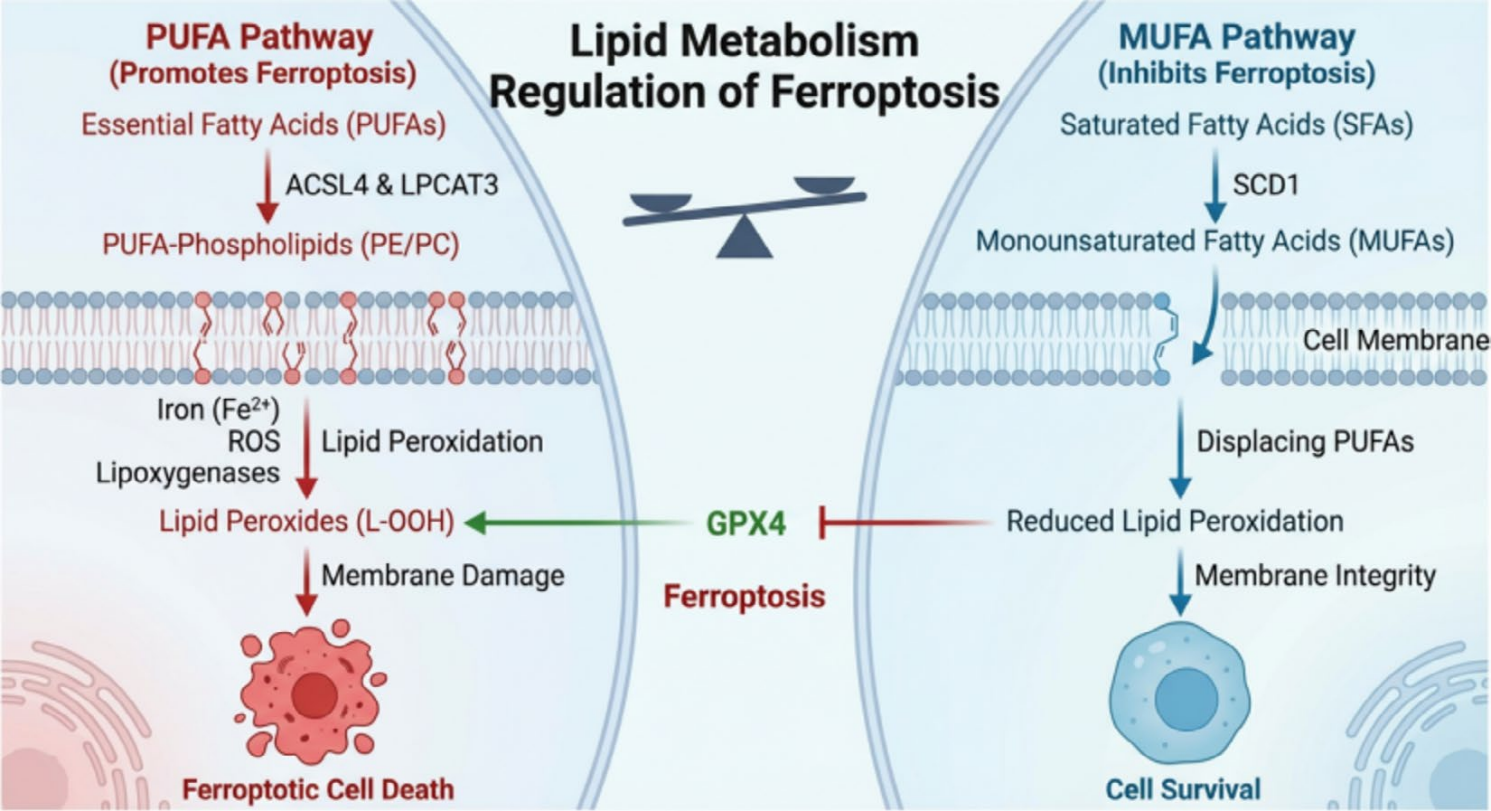
Regulation of ferroptosis by lipid metabolism. Left Panel (PUFA Pathway): PUFAs are incorporated into cellular membrane phospholipids, such as PE and PC, by enzymes including ACSL4 and LPCAT3. In the presence of iron (Fe2+), ROS, and lipoxygenases, these PUFA‐containing phospholipids undergo lipid peroxidation, generating lipid hydroperoxides (L‐OOH). Accumulation of L‐OOH, particularly when GPX4 activity is insufficient, leads to membrane damage and ultimately executes ferroptotic cell death. Right Panel (MUFA Pathway): SFAs are converted into MUFAs by the enzyme SCD1. These MUFAs are subsequently incorporated into cell membranes, where they displace PUFAs. As MUFAs are less susceptible to peroxidation, this process reduces the membrane's sensitivity to lipid peroxidation, maintains membrane integrity, and thereby inhibits ferroptosis, promoting cell survival. Center: The balance between these two pathways is represented by a central scale, illustrating “Lipid Metabolism Regulation.” GPX4 acts as a critical antioxidant enzyme, preventing PUFA‐driven ferroptosis by neutralizing L‐OOH. The MUFA pathway indirectly supports GPX4 function by reducing the generation of L‐OOH, collectively determining the cell's fate.

Lipid metabolism dictates ferroptosis sensitivity through the balance of membrane fatty acid composition. PUFAs, particularly when esterified into phospholipids (e.g., PE and PC) by enzymes like ACSL4 and LPCAT3, are the obligate substrates for lethal lipid peroxidation driven by iron and lipoxygenases (Figure 4, Left Panel) [40, 41, 42]. Dysregulation of PUFA incorporation sensitizes cells to oxidative damage. Conversely, MUFAs exert a protective effect. Synthesized primarily by SCD1, MUFAs displace PUFAs in membrane phospholipids, reducing the pool of peroxidizable substrates and maintaining membrane integrity (Figure 4, Right Panel) [28, 43]. This dynamic interplay acts as a metabolic scale: enhancing MUFA synthesis via pathways like C12ORF49/SCD1 inhibits ferroptosis, while PUFA accumulation promotes it. Thus, targeting this lipid balance represents a critical therapeutic strategy [1, 44]. Additionally, lipid transporters such as SLC47A1 have been implicated in maintaining MUFA‐enriched lipid pools that confer resistance [45, 46].

### 3.3 Key Molecules and Signaling Pathways Regulating Ferroptosis

**FIGURE 5:**
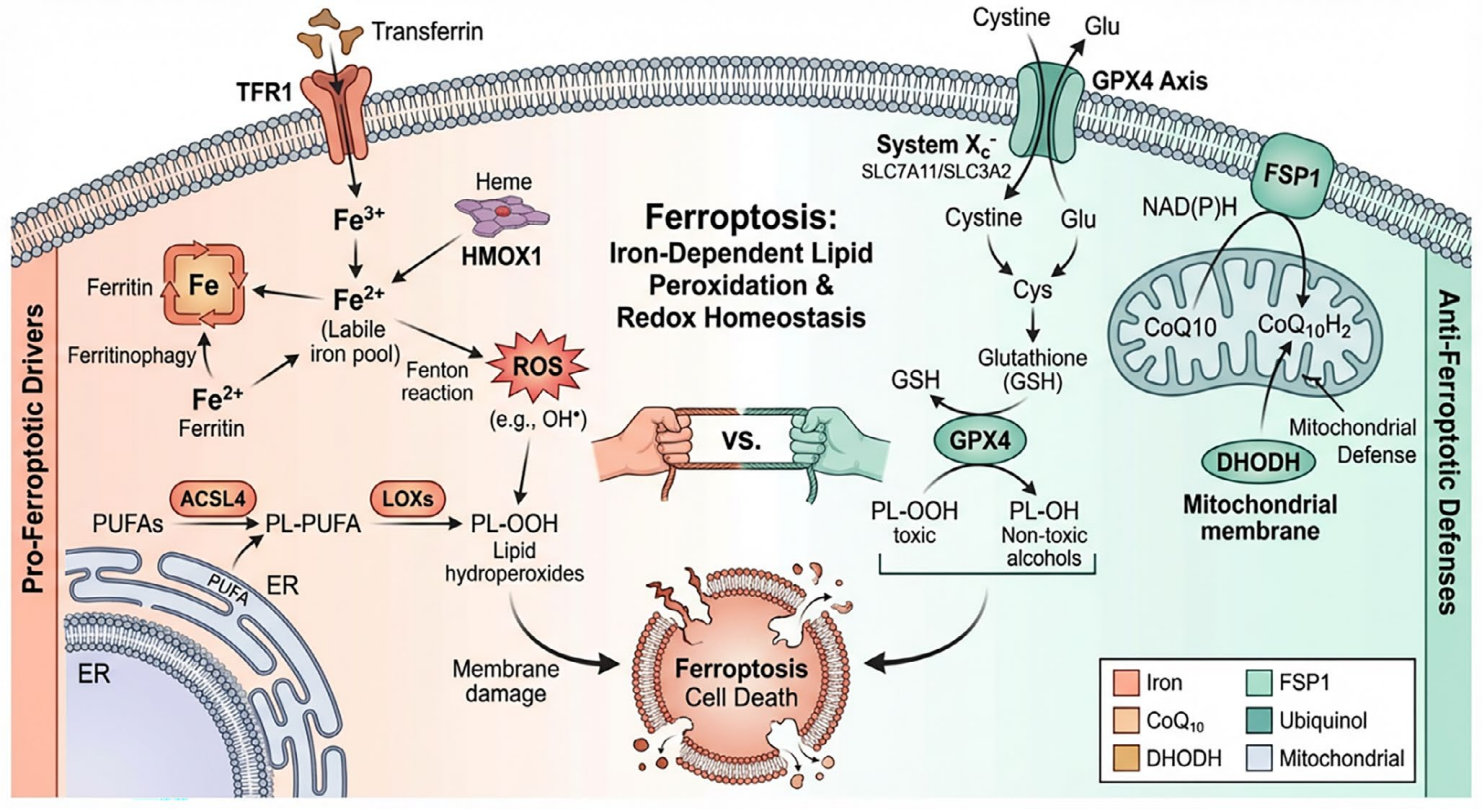
Schematic representation of key molecules and signaling pathways regulating ferroptosis. Ferroptosis is driven by iron‐dependent lipid peroxidation and is governed by a complex interplay of three main regulatory axes: antioxidant defense systems, iron metabolism, and lipid metabolic pathways. (A) Antioxidant defense: Cellular survival is maintained by restricting lipid peroxidation. The canonical pathway involves System Xc‐ (comprising SLC7A11 and SLC3A2 subunits), which imports cystine to synthesize GSH. GSH is the essential cofactor for GPX4, a selenoenzyme that directly reduces toxic lipid hydroperoxides (Lipid‐OOH) to non‐toxic lipid alcohols (Lipid‐OH). Parallel to GPX4, FSP1 functions on the plasma membrane, utilizing NAD(P)H to regenerate ubiquinol (reduced CoQ10), a potent lipophilic antioxidant that suppresses membrane lipid peroxidation. Mitochondrial DHODH also contributes to maintaining cellular redox homeostasis. (B) Iron metabolism: Intracellular labile iron (Fe^2+^) catalyzes the Fenton reaction to generate ROS, which initiate lipid peroxidation. Iron levels are increased by TFR1‐mediated uptake, HMOX1‐dependent heme degradation, and ferritin degradation via ferritinophagy. Conversely, iron storage by ferritin and export via FPN reduce the labile iron pool, inhibiting ferroptosis. (C) Lipid metabolism: The sensitivity to ferroptosis is dictated by the lipid composition of membranes. ACSL4 enriches membranes with PUFAs, which are preferentially oxidized by lipoxygenases (LOXs) and iron‐derived ROS to generate lethal Lipid‐OOH. The accumulation of these lipid peroxides, when unchecked by antioxidant defenses, triggers ferroptotic cell death. ACSL4, Acyl‐CoA synthetase long‐chain family member 4; CoQ10, Coenzyme Q10; DHODH, dihydroorotate dehydrogenase; FPN, ferroportin; FSP1, ferroptosis suppressor protein 1; GPX4, glutathione peroxidase 4; GSH, glutathione; HMOX1, heme oxygenase‐1; LOXs, lipoxygenases; PUFA, polyunsaturated fatty acid; ROS, reactive oxygen species; TFR1, transferrin receptor 1.

Ferroptosis is governed by a complex network involving antioxidant defenses, iron metabolism, and lipid regulation (Figure 5). The primary defense is the System Xc^−^/GSH/GPX4 axis; GPX4 utilizes glutathione to reduce toxic lipid hydroperoxides to non‐toxic alcohols. Parallel independent systems, such as the FSP1‐CoQ10 axis and mitochondrial DHODH, further bolster antioxidant capacity [47, 48, 49]. On the other hand, iron metabolism proteins (TFR1, Ferritin, FPN, HMOX1) regulate the labile iron pool required for the Fenton reaction, which generates the ROS initiating lipid peroxidation [50, 51]. Finally, lipid metabolic enzymes like ACSL4 and lipoxygenases (LOXs) actively drive the ferroptotic process by enriching membranes with oxidizable PUFAs. The integration of these signals determines cell fate, with C12ORF49 intervening specifically at the level of lipid composition [49, 52, 53, 54].

## 4 The Crosstalk Regulatory Mechanism Between C12ORF49 and Ferroptosis

### 4.1 C12ORF49 Inhibits Ferroptosis Through the SREBP1/SCD1 Molecular Mechanism

**FIGURE 6:**
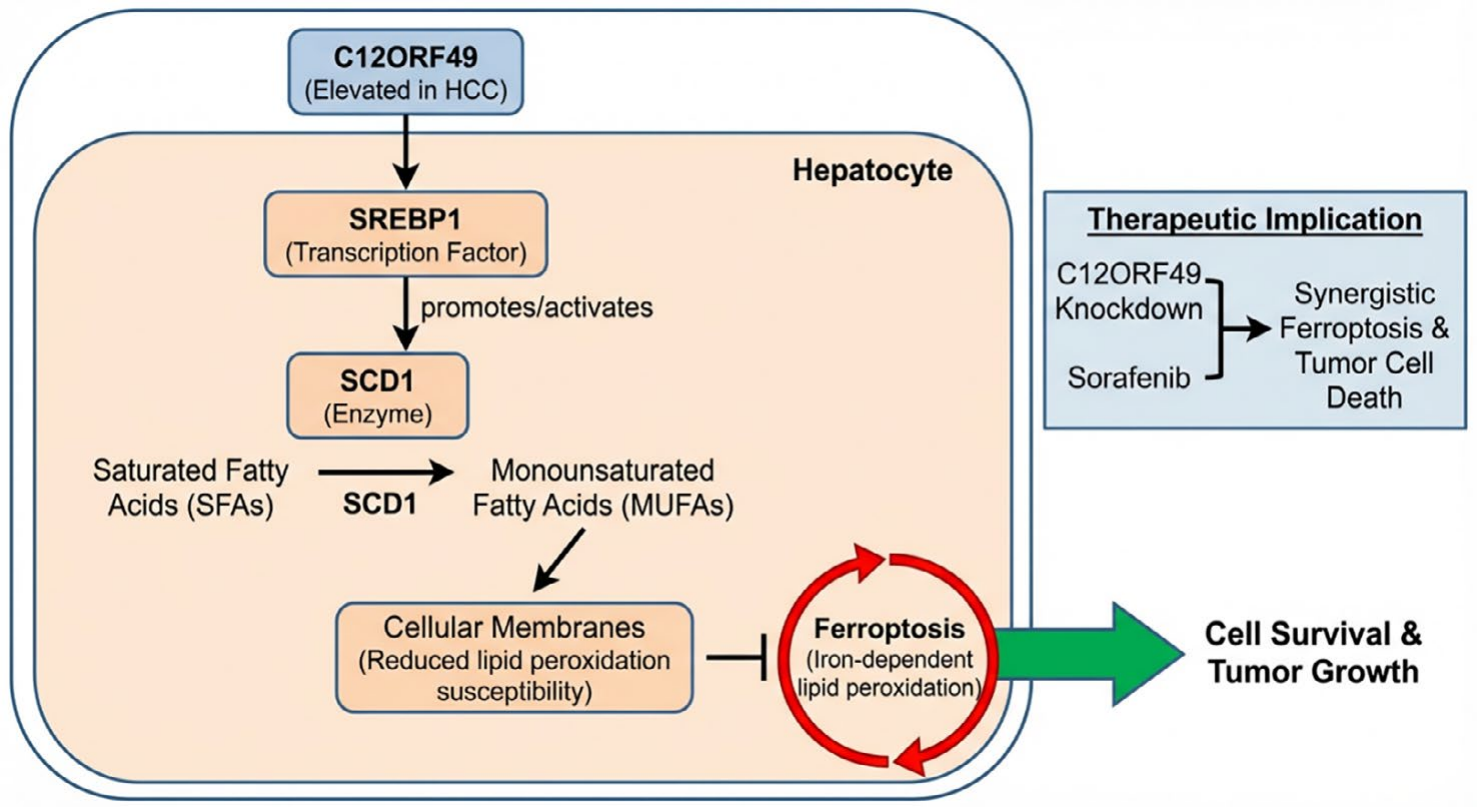
C12ORF49 promotes HCC cell survival and tumor growth by inhibiting ferroptosis through the SREBP1/SCD1‐mediated lipid desaturation pathway. The diagram illustrates the molecular mechanism within a hepatocyte. C12ORF49, which is elevated in HCC, activates the transcription factor SREBP1. SREBP1 then promotes the activity of the enzyme SCD1. SCD1 catalyzes the conversion of SFAs into MUFAs. The incorporation of these MUFAs into cellular membranes reduces their susceptibility to lipid peroxidation, thereby inhibiting ferroptosis (iron‐dependent lipid peroxidation) and ultimately fostering cell survival and tumor growth.

In HCC, C12ORF49 functions as a potent ferroptosis suppressor by rewiring the SREBP1/SCD1 signaling axis. Mechanistically, C12ORF49 promotes the nuclear translocation of SREBP1, which transcriptionally activates SCD1. The resulting SCD1 enzyme converts SFAs into MUFAs, which are then incorporated into cellular membranes (Figure 6). This lipid remodeling significantly reduces the abundance of peroxidizable PUFA‐phospholipids, thereby creating a “ferroptosis‐resistant” membrane phenotype. Experimental evidence shows that C12ORF49 knockdown reverses this process, leading to lipid peroxide accumulation and halted tumor growth, while its overexpression confers robust protection against oxidative stress [28]. This regulatory cascade positions C12ORF49 as a pivotal upstream switch that cancer cells exploit to evade ferroptotic death.

### 4.2 Impact of C12ORF49 Expression Changes on Ferroptosis Sensitivity in HCC

**FIGURE 7:**
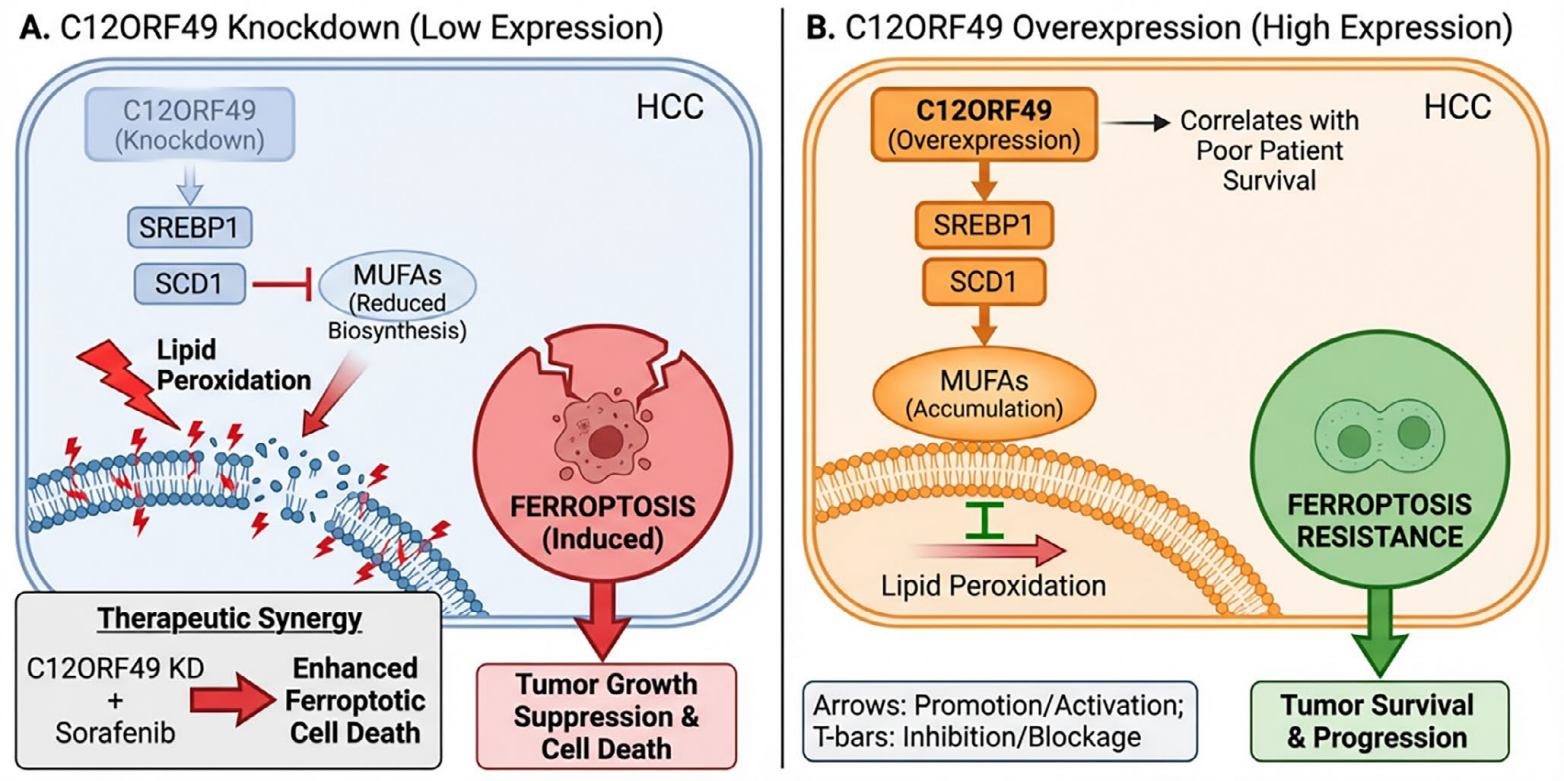
Schematic illustration depicting the impact of C12ORF49 expression dynamics on ferroptosis sensitivity and tumor fate in HCC. (A) C12ORF49 knockdown impairs the SREBP1/SCD1 signaling axis, leading to reduced biosynthesis of MUFAs. The insufficient supply of MUFAs renders cellular membranes highly susceptible to lipid peroxidation, thereby inducing ferroptosis and resulting in suppressed tumor growth and cell death. The bottom‐left inset highlights the therapeutic synergy where combining C12ORF49 knockdown with Sorafenib significantly enhances ferroptotic cell death. (B) C12ORF49 overexpression, which clinically correlates with poor patient survival, hyperactivates SREBP1 and SCD1. This leads to the accumulation of MUFAs that integrate into cellular membranes, effectively inhibiting lipid peroxidation. Consequently, HCC cells develop resistance to ferroptosis, promoting tumor survival and progression.

The expression levels of C12ORF49 directly dictate the ferroptotic threshold of HCC cells (Figure 7). Silencing C12ORF49 disrupts SREBP1/SCD1 signaling, collapsing MUFA biosynthesis and forcing the membrane composition towards a PUFA‐rich state, which renders cells highly susceptible to peroxidation (Figure 7A). Conversely, high C12ORF49 levels, correlating with poor clinical survival, hyperactivate MUFA synthesis to buffer oxidative insults and facilitate tumor progression. (Figure 7B) [28].

Crucially, the regulatory impact of C12ORF49 is likely modulated by the distinct etiological landscapes and heterogeneity inherent to HCC. In viral‐associated HCC, particularly Hepatitis B Virus (HBV) infection, viral proteins, such as HBx have been implicated in hijacking C12ORF49 signaling to facilitate immune evasion and metabolic adaptation, as evidenced by the KLF16‐mediated PD‐L1 axis [30]. Conversely, in the context of metabolic dysfunction‐associated steatohepatitis (MASH)‐driven HCC, the chronic lipid‐rich environment may necessitate constitutive upregulation of C12ORF49 to manage lipotoxicity and support membrane integrity via SREBP‐dependent mechanisms. Furthermore, disease staging appears to influence C12ORF49 dependency; advanced tumors, often characterized by hypoxia and heightened biosynthetic demands, may rely more heavily on C12ORF49‐driven de novo lipogenesis to avert ferroptosis compared to early‐stage lesions. This variability suggests that C12ORF49's function is context‐dependent, and stratifying patients based on viral status, metabolic background, and disease stage will be essential for optimizing targeted therapies.

## 5 Synergistic Effects of C12ORF49 Combined with Sorafenib Treatment

### 5.1 Sorafenib's Antitumor Mechanism and Its Limitations

Sorafenib, the standard first‐line systemic therapy for advanced HCC, exerts antitumor effects through dual mechanisms: inhibition of tumor proliferation via the RAF/MEK/ERK pathway and suppression of angiogenesis by targeting VEGFRs and PDGFRs [55, 56]. Additionally, it exerts immunomodulatory effects by reprogramming the tumor immune microenvironment, showing potential synergy with immune agonists [57]. However, its clinical efficacy is modest, extending median survival by only 3–5 months due to complex resistance mechanisms [58].

Resistance to Sorafenib is multifactorial. Intrinsic and acquired resistance often involve the activation of alternative signaling pathways (e.g., PI3K/AKT/mTOR) and epithelial‐mesenchymal transition (EMT) [59, 60]. Critically, metabolic reprogramming plays a central role; for instance, FASN upregulation antagonizes Sorafenib‐induced ferroptosis by stabilizing HIF1α and SLC7A11 [61]. Furthermore, Sorafenib can induce adaptive translation reprogramming to sustain survival gene expression [62]. Beyond cellular mechanisms, the immune microenvironment contributes significantly to resistance. Tumor‐associated macrophages (TAMs) secrete miRNAs to activate survival signals [63], while myeloid‐derived suppressor cells (MDSCs) promote fibrosis and impair drug efficacy [64].

Clinical utility is further hampered by significant toxicity (e.g., hand‐foot reaction) and pharmacokinetic limitations such as poor bioavailability [55]. While novel nanotechnology‐based delivery systems show promise in improving safety and retention [65, 66, 67], combination therapies remain the most viable strategy to overcome resistance. Current explorations include combining Sorafenib with oxidative stress modulators (artesunate, ursolic acid, curcumin) [68, 69, 70, 71], specific molecular inhibitors targeting resistance mediators (YAP, KIF14, USP22) [72, 73, 74] or immune checkpoint inhibitors like camrelizumab [75]. These challenges underscore the urgent need for novel targets like C12ORF49 that can specifically dismantle metabolic resistance.

### 5.2 C12ORF49 Suppression Enhances Sorafenib‐Induced Ferroptosis

Recent studies identify C12ORF49 inhibition as a potent strategy to overcome Sorafenib resistance. Sorafenib inherently promotes ferroptosis by depleting GSH and accumulating ROS; however, this effect is frequently blunted by compensatory lipid remodeling in tumors. Experimental evidence demonstrates that C12ORF49 knockdown synergistically amplifies Sorafenib efficacy. In vitro, silencing C12ORF49 significantly enhances Sorafenib‐induced lipid peroxidation and iron accumulation. In vivo, the combination yields marked tumor regression compared to monotherapy [28]. Mechanistically, this synergy is driven by the dismantling of the “MUFA shield.” By inhibiting C12ORF49, the protective metabolic buffer is removed, forcing the membrane lipid composition towards a PUFA‐rich, peroxidation‐prone state. Consequently, when combined with the oxidative stress induced by Sorafenib, C12ORF49 suppression lowers the threshold for lethal ferroptosis (Figure 7A, inset) [28].

However, acknowledging translational barriers is essential for a realistic clinical perspective. Despite compelling preclinical data, targeting C12ORF49 in patients presents substantial challenges. First, as a Golgi‐resident protein, C12ORF49 currently lacks specific small‐molecule inhibitors, raising questions regarding its “druggability.” Second, given its fundamental role in global lipid homeostasis, systemic inhibition carries risks of on‐target toxicity in healthy lipid‐active tissues like the liver. This necessitates the development of tumor‐specific delivery systems. Furthermore, due to HCC heterogeneity, not all tumors may rely on C12ORF49‐driven lipogenesis, underscoring the need for predictive biomarkers to stratify patients likely to benefit from this combinatorial strategy.

## 6 Conclusion

In conclusion, the emerging role of C12ORF49 as a pivotal regulator of lipid metabolism and ferroptosis in HCC underscores its significance in tumor biology. From an expert perspective, the current body of evidence delineates how C12ORF49 modulates the SREBP1/SCD1 signaling axis to suppress ferroptotic cell death, revealing the sophisticated metabolic adaptations cancer cells exploit to navigate the hostile tumor microenvironment.

A critical frontier for future research lies in elucidating the precise molecular crosstalk between C12ORF49 and the canonical ferroptosis executioners, particularly GPX4 and ACSL4. C12ORF49 likely functions as a strategic metabolic checkpoint, potentially remodeling the membrane phospholipid landscape to limit the incorporation of PUFAs—the obligate substrates for ACSL4‐mediated peroxidation—or conversely, reinforcing the antioxidant shield by stabilizing GPX4. Unraveling whether C12ORF49 acts as an upstream “metabolic rheostat” that dampens ACSL4 activity while boosting GPX4 efficiency will be pivotal for mapping the hierarchy of ferroptotic resistance in HCC.

Translationally, the correlation between elevated C12ORF49 expression and poor clinical outcomes, coupled with the synergistic antitumor effects observed with combined C12ORF49 knockdown and sorafenib treatment, validates its potential to overcome drug resistance. To harness this potential, future studies must prioritize robust validation in clinically relevant models, such as patient‐derived xenografts (PDX) or organoids. The ultimate goal is to establish C12ORF49 not merely as a prognostic indicator, but as a specific predictive biomarker that identifies patient stratifications where C12ORF49‐driven lipogenesis is the dominant survival mechanism.

In summary, targeting C12ORF49 could dismantle the “metabolic fortress” of HCC cells, re‐sensitizing them to ferroptosis‐inducing agents. By harmonizing these mechanistic insights with rigorous clinical validation, we can unlock new dimensions for precision oncology, ultimately contributing to the development of more effective, personalized treatment strategies for patients afflicted with hepatocellular carcinoma.

## Funding

This work was supported by the Natural Science Foundation of Inner Mongolia Autonomous Region (2025ZD009).

## Disclosure

During the preparation of this manuscript, the authors used an artificial intelligence–based tool (Gemini 3, Google) to assist in the generation of schematic figures for illustrative and visualization purposes only. The scientific concepts, content accuracy, and interpretations presented in the figures were fully conceived, reviewed, and approved by the authors. The figures do not represent original experimental data, patient data, or previously published materials, and the authors take full responsibility for the content of the figures.

## Ethics Statement

The authors have nothing to report.

## Consent

The authors have nothing to report.

## Conflicts of Interest

The authors declare no conflicts of interest.

## Data Availability

Data sharing not applicable to this article as no datasets were generated or analysed during the current study.

## References

[1] Y. Sun , Z. Xue , T. Huang , X. Che , and G. Wu , “Lipid Metabolism in Ferroptosis and Ferroptosis‐Based Cancer Therapy,” Frontiers in Oncology 12 (2022): 941618, 10.3389/fonc.2022.941618. 35978815 PMC9376317

[2] L. Zeng , X. Liu , C. Geng , X. Gao , and L. Liu , “Ferroptosis in Cancer (Review),” Oncology Letters 28, no. 1 (2024): 304, 10.3892/ol.2024.14437. 38774452 PMC11106693

[3] C. M. Bebber , F. Müller , L. Prieto Clemente , J. Weber , and S. von Karstedt , “Ferroptosis in Cancer Cell Biology,” Cancers (Basel) 12, no. 1 (2020): 164, 10.3390/cancers12010164. 31936571 PMC7016816

[4] T. Nakamura and M. Conrad , “Exploiting Ferroptosis Vulnerabilities in Cancer,” Nature Cell Biology 26, no. 9 (2024): 1407–1419, 10.1038/s41556-024-01425-8. 38858502

[5] L. Zhao , X. Zhou , F. Xie , et al., “Ferroptosis in Cancer and Cancer Immunotherapy,” Cancer Commun (Lond) 42, no. 2 (2022): 88–116, 10.1002/cac2.12250. 35133083 PMC8822596

[6] A. Wahida and M. Conrad , “Decoding Ferroptosis for Cancer Therapy,” Nature Reviews. Cancer 25, no. 12 (2025): 910–924, 10.1038/s41568-025-00864-1. 41073537

[7] R. Zhang , J. Chen , S. Wang , W. Zhang , Q. Zheng , and R. Cai , “Ferroptosis in Cancer Progression,” Cells 12, no. 14 (2023): 1820, 10.3390/cells12141820. 37508485 PMC10378139

[8] J. Yin , X. Meng , L. Peng , et al., “Ferroptosis and Cancer Immunotherapy,” Current Molecular Medicine 23, no. 5 (2023): 401–409, 10.2174/1566524022666220509124608. 35579155

[9] S. Zheng and X. Y. Guan , “Ferroptosis: Promising Approach for Cancer and Cancer Immunotherapy,” Cancer Letters 561 (2023): 216152, 10.1016/j.canlet.2023.216152. 37023938

[10] H. Li , S. Li , Y. Kanamori , S. Liu , and T. Moroishi , “Auranofin Resensitizes Ferroptosis‐Resistant Lung Cancer Cells to Ferroptosis Inducers,” Biochemical and Biophysical Research Communications 770 (2025): 151992, 10.1016/j.bbrc.2025.151992. 40373379

[11] C. Li , F. Wang , L. Cui , S. Li , J. Zhao , and L. Liao , “Association Between Abnormal Lipid Metabolism and Tumor,” Frontiers in Endocrinology (Lausanne) 14 (2023): 1134154, 10.3389/fendo.2023.1134154. PMC10248433 37305043

[12] R. Vishwa , B. BharathwajChetty , S. Girisa , et al., “Lipid Metabolism and Its Implications in Tumor Cell Plasticity and Drug Resistance: What We Learned Thus Far?,” Cancer Metastasis Reviews 43, no. 1 (2024): 293–319, 10.1007/s10555-024-10170-1. 38438800

[13] W. Liu , S. Dong , F. Hao , Y. Gao , and Q. Wei , “Lipid Metabolic Reprogramming in Colorectal Cancer: Mechanisms and Therapeutic Strategies,” Frontiers in Immunology 16 (2025): 1603032, 10.3389/fimmu.2025.1603032. 40718481 PMC12289696

[14] S. Chaudhry , S. N. Thomas , and G. E. Simmons, Jr. , “Targeting Lipid Metabolism in the Treatment of Ovarian Cancer,” Oncotarget 13 (2022): 768–783, 10.18632/oncotarget.28241. 35634242 PMC9132258

[15] X. Xie , L. Tian , Y. Zhao , et al., “BACH1‐Induced Ferroptosis Drives Lymphatic Metastasis by Repressing the Biosynthesis of Monounsaturated Fatty Acids,” Cell Death & Disease 14, no. 1 (2023): 48, 10.1038/s41419-023-05571-z. 36670112 PMC9860034

[16] J. Rodencal and S. J. Dixon , “A Tale of Two Lipids: Lipid Unsaturation Commands Ferroptosis Sensitivity,” Proteomics 23, no. 6 (2023): e2100308, 10.1002/pmic.202100308. 36398995

[17] K. Shan , G. Fu , J. Li , et al., “Cis‐Monounsaturated Fatty Acids Inhibit Ferroptosis Through Downregulation of Transferrin Receptor 1,” Nutrition Research 118 (2023): 29–40, 10.1016/j.nutres.2023.07.002. 37544230

[18] J. Lee and J. L. Roh , “Lipid Metabolism in Ferroptosis: Unraveling Key Mechanisms and Therapeutic Potential in Cancer,” Biochimica et Biophysica Acta. Reviews on Cancer 1880, no. 1 (2025): 189258, 10.1016/j.bbcan.2024.189258. 39746458

[19] U. Sen , C. Coleman , and T. Sen , “Stearoyl Coenzyme A Desaturase‐1: Multitasker in Cancer, Metabolism, and Ferroptosis,” Trends Cancer 9, no. 6 (2023): 480–489, 10.1016/j.trecan.2023.03.003. 37029018

[20] I. Koeken , M. Walravens , R. Fernández‐Acosta , et al., “Dual Lipid Modulation Overcomes Ferroptosis Resistance in High‐Risk Neuroblastoma,” Cell Death and Differentiation (2025), 10.1038/s41418-025-01623-3. PMC13156318 41299087

[21] Z. Wang , Y. Wang , Z. Li , W. Xue , S. Hu , and X. Kong , “Lipid Metabolism as a Target for Cancer Drug Resistance: Progress and Prospects,” Frontiers in Pharmacology 14 (2023): 1274335, 10.3389/fphar.2023.1274335. 37841917 PMC10571713

[22] N. Germain , M. Dhayer , M. Boileau , Q. Fovez , J. Kluza , and P. Marchetti , “Lipid Metabolism and Resistance to Anticancer Treatment,” Biology‐Basel 9, no. 12 (2020): 474, 10.3390/biology9120474. 33339398 PMC7766644

[23] J. Xiao , Y. Xiong , L. T. Yang , et al., “POST1/C12ORF49 Regulates the SREBP Pathway by Promoting Site‐1 Protease Maturation,” Protein & Cell 12, no. 4 (2021): 279–296, 10.1007/s13238-020-00753-3. 32666500 PMC8019017

[24] E. C. Bayraktar , K. La , K. Karpman , et al., “Metabolic Coessentiality Mapping Identifies C12orf49 as a Regulator of SREBP Processing and Cholesterol Metabolism,” Nature Metabolism 2, no. 6 (2020): 487–498, 10.1038/s42255-020-0206-9. PMC7384252 32694732

[25] A. Loregger , M. Raaben , J. Nieuwenhuis , et al., “Haploid Genetic Screens Identify SPRING/C12ORF49 as a Determinant of SREBP Signaling and Cholesterol Metabolism,” Nature Communications 11, no. 1 (2020): 1128, 10.1038/s41467-020-14811-1. PMC7048761 32111832

[26] S. Hendrix and N. Zelcer , “A New SPRING in Lipid Metabolism,” Current Opinion in Lipidology 34, no. 5 (2023): 201–207, 10.1097/MOL.0000000000000894. 37548386

[27] I. Micallo , A. V. Bullington , D. L. Kober , and N. Zelcer , “SPRINGing Off the Lock: The Role of SPRING in S1P Activity and SREBP‐Regulated Lipid Metabolism,” Current Opinion in Lipidology 36, no. 5 (2025): 276–283, 10.1097/MOL.0000000000001003. 40747999 PMC12419022

[28] H. C. Yu , L. Jin , L. Bai , Y. J. Zhang , and Z. X. Yang , “C12ORF49 Inhibits Ferroptosis in Hepatocellular Carcinoma Cells via Reprogramming SREBP1/SCD1‐Mediated Lipid Metabolism,” Cell Death Discov 11, no. 1 (2025): 178, 10.1038/s41420-025-02480-2. 40240331 PMC12003882

[29] F. Zhang , Z. Wu , Y. Xiang , et al., “SOX4 Reprograms Fatty Acid Metabolism Through the CHREBP to Inhibit Ferroptosis in Hepatocellular Carcinoma,” Cell Death Discovery 11, no. 1 (2025): 246, 10.1038/s41420-025-02527-4. 40399256 PMC12095664

[30] W. Chen , C. Lin , L. Wang , et al., “Hepatitis B Virus X Protein Promotes the Progression and Immune Escape of Hepatocellular Carcinoma by Activating KLF16‐C12orf49‐PD‐L1 Axis,” Oncogene 44, no. 48 (2025): 4747–4762, 10.1038/s41388-025-03625-4. 41238928

[31] Y. Tao , J. Luo , H. Zhu , Y. Chu , and L. Pei , “Chromosome 12 Open Reading Frame 49 Promotes Tumor Growth and Predicts Poor Prognosis in Colorectal Cancer,” Digestive Diseases and Sciences 68, no. 4 (2023): 1306–1315, 10.1007/s10620-022-07751-x. 36348128 PMC10102024

[32] Y. Shinden , T. Hirashima , N. Nohata , et al., “Molecular Pathogenesis of Breast Cancer: Impact of miR‐99a‐5p and miR‐99a‐3p Regulation on Oncogenic Genes,” Journal of Human Genetics 66, no. 5 (2021): 519–534, 10.1038/s10038-020-00865-y. 33177704

[33] S. Hendrix , J. M. E. Tan , K. Ndoj , et al., “SPRING Is a Dedicated Licensing Factor for SREBP‐Specific Activation by S1P,” Molecular and Cellular Biology 44, no. 4 (2024): 123–137, 10.1080/10985549.2024.2348711. 38747374 PMC11110692

[34] A. F. Flórez and H. Alborzinia , “Ferroptosis: Concepts and Definitions,” Advances in Experimental Medicine and Biology 1301 (2021): 1–5, 10.1007/978-3-030-62026-4_1. 34370284

[35] J. Lei , Z. Chen , S. Song , C. Sheng , S. Song , and J. Zhu , “Insight Into the Role of Ferroptosis in Non‐Neoplastic Neurological Diseases,” Frontiers in Cellular Neuroscience 14 (2020): 231, 10.3389/fncel.2020.00231. 32848622 PMC7424047

[36] X. Zhang and X. Li , “Abnormal Iron and Lipid Metabolism Mediated Ferroptosis in Kidney Diseases and Its Therapeutic Potential,” Metabolites 12, no. 1 (2022): 58, 10.3390/metabo12010058. 35050181 PMC8779729

[37] F. Wang , J. He , R. Xing , T. Sha , and B. Sun , “Molecular Mechanisms of Ferroptosis and Their Role in Inflammation,” International Reviews of Immunology 42, no. 1 (2023): 71–81, 10.1080/08830185.2021.2016739. 34918993

[38] Y. Kong , J. Li , R. Lin , et al., “Understanding the Unique Mechanism of Ferroptosis: A Promising Therapeutic Target,” Frontiers in Cell and Development Biology 11 (2023): 1329147, 10.3389/fcell.2023.1329147. PMC10982331 38562992

[39] X. Chen , A. S. Tsvetkov , H. M. Shen , et al., “International Consensus Guidelines for the Definition, Detection, and Interpretation of Autophagy‐Dependent Ferroptosis,” Autophagy 20, no. 6 (2024): 1213–1246, 10.1080/15548627.2024.2319901. 38442890 PMC11210914

[40] J. Y. Lee , W. K. Kim , K. H. Bae , S. C. Lee , and E. W. Lee , “Lipid Metabolism and Ferroptosis,” Biology‐Basel 10, no. 3 (2021): 184, 10.3390/biology10030184. 33801564 PMC8000263

[41] Z. Lin , J. Liu , R. Kang , M. Yang , and D. Tang , “Lipid Metabolism in Ferroptosis,” Advanced Biology 5, no. 8 (2021): e2100396, 10.1002/adbi.202100396. 34015188

[42] L. E. Pope and S. J. Dixon , “Regulation of Ferroptosis by Lipid Metabolism,” Trends in Cell Biology 33, no. 12 (2023): 1077–1087, 10.1016/j.tcb.2023.05.003. 37407304 PMC10733748

[43] J. W. Kim , J. Y. Lee , M. Oh , and E. W. Lee , “An Integrated View of Lipid Metabolism in Ferroptosis Revisited via Lipidomic Analysis,” Experimental & Molecular Medicine 55, no. 8 (2023): 1620–1631, 10.1038/s12276-023-01077-y. 37612411 PMC10474074

[44] D. Sun , L. Wang , Y. Wu , et al., “Lipid Metabolism in Ferroptosis: Mechanistic Insights and Therapeutic Potential,” Frontiers in Immunology 16 (2025): 1545339, 10.3389/fimmu.2025.1545339. 40134420 PMC11932849

[45] Z. Lin , J. Liu , F. Long , et al., “The Lipid Flippase SLC47A1 Blocks Metabolic Vulnerability to Ferroptosis,” Nature Communications 13, no. 1 (2022): 7965, 10.1038/s41467-022-35707-2. PMC9794750 36575162

[46] D. Liang , A. M. Minikes , and X. Jiang , “Ferroptosis at the Intersection of Lipid Metabolism and Cellular Signaling,” Molecular Cell 82, no. 12 (2022): 2215–2227, 10.1016/j.molcel.2022.03.022. 35390277 PMC9233073

[47] W. Li , L. Liang , S. Liu , H. Yi , and Y. Zhou , “FSP1: A Key Regulator of Ferroptosis,” Trends in Molecular Medicine 29, no. 9 (2023): 753–764, 10.1016/j.molmed.2023.05.013. 37357101

[48] X. Chen , J. Li , R. Kang , D. J. Klionsky , and D. Tang , “Ferroptosis: Machinery and Regulation,” Autophagy 17, no. 9 (2021): 2054–2081, 10.1080/15548627.2020.1810918. 32804006 PMC8496712

[49] H. Liao , J. Shi , K. Wen , et al., “Molecular Targets of Ferroptosis in Hepatocellular Carcinoma,” Journal of Hepatocellular Carcinoma 8 (2021): 985–996, 10.2147/JHC.S325593. 34466409 PMC8403010

[50] J. Yin , J. Zhan , Q. Hu , S. Huang , and W. Lin , “Fluorescent Probes for Ferroptosis Bioimaging: Advances, Challenges, and Prospects,” Chemical Society Reviews 52, no. 6 (2023): 2011–2030, 10.1039/d2cs00454b. 36880388

[51] Z. Wang , “Iron Regulation in Ferroptosis,” Nature Cell Biology 25, no. 4 (2023): 515, 10.1038/s41556-023-01129-5. 37059879

[52] J. Liu , R. Kang , and D. Tang , “Signaling Pathways and Defense Mechanisms of Ferroptosis,” FEBS Journal 289, no. 22 (2022): 7038–7050, 10.1111/febs.16059. 34092035

[53] B. Gan , “Mitochondrial Regulation of Ferroptosis,” Journal of Cell Biology 220, no. 9 (2021): e202105043, 10.1083/jcb.202105043. 34328510 PMC8329737

[54] X. Tang , Y. Niu , J. Jian , et al., “Potential Applications of Ferroptosis Inducers and Regulatory Molecules in Hematological Malignancy Therapy,” Critical Reviews in Oncology/Hematology 193 (2023): 104203, 10.1016/j.critrevonc.2023.104203. 37979734

[55] Y. Pang , A. Eresen , Z. Zhang , et al., “Adverse Events of Sorafenib in Hepatocellular Carcinoma Treatment,” American Journal of Cancer Research 12, no. 6 (2022): 2770–2782. 35812068 PMC9251699

[56] W. Zhang , X. Hong , Y. Xiao , H. Wang , and X. Zeng , “Sorafenib Resistance and Therapeutic Strategies in Hepatocellular Carcinoma,” Biochimica et Biophysica Acta ‐ Reviews on Cancer 1880, no. 3 (2025): 189310, 10.1016/j.bbcan.2025.189310. 40187502

[57] Y. He , L. Zhan , J. Shi , et al., “The Combination of R848 With Sorafenib Enhances Antitumor Effects by Reprogramming the Tumor Immune Microenvironment and Facilitating Vascular Normalization in Hepatocellular Carcinoma,” Advanced Science 10, no. 18 (2023): e2207650, 10.1002/advs.202207650. 37083239 PMC10288281

[58] Y. Liang , “Mechanisms of Sorafenib Resistance in Hepatocellular Carcinoma,” Clinical Research in Hepatology and Gastroenterology 48, no. 8 (2024): 102434, 10.1016/j.clinre.2024.102434. 39084553

[59] R. Tong , X. Feng , J. Sun , et al., “Co‐Delivery of siNRF2 and Sorafenib by a “Click” Dual Functioned Hyperbranched Nanocarrier for Synergistically Inducing Ferroptosis in Hepatocellular Carcinoma,” Small 20, no. 21 (2024): e2307273, 10.1002/smll.202307273. 38102096

[60] R. Zheng , S. Weng , J. Xu , et al., “Autophagy and Biotransformation Affect Sorafenib Resistance in Hepatocellular Carcinoma,” Computational and Structural Biotechnology Journal 21 (2023): 3564–3574, 10.1016/j.csbj.2023.07.005. 37520282 PMC10372478

[61] Y. Li , W. Yang , Y. Zheng , et al., “Targeting Fatty Acid Synthase Modulates Sensitivity of Hepatocellular Carcinoma to Sorafenib via Ferroptosis,” Journal of Experimental & Clinical Cancer Research 42, no. 1 (2023): 6, 10.1186/s13046-022-02567-z. 36604718 PMC9817350

[62] L. Contreras , A. Rodríguez‐Gil , J. Muntané , and J. de la Cruz , “Sorafenib‐Associated Translation Reprogramming in Hepatocellular Carcinoma Cells,” RNA Biology 22, no. 1 (2025): 1–11, 10.1080/15476286.2025.2483484. PMC11934173 40116042

[63] W. Li , B. Zhao , Q. Wang , J. Lu , X. Wu , and X. Chen , “M2 Macrophage Exosomes Promote Resistance to Sorafenib in Hepatocellular Carcinoma Cells via miR‐200c‐3p,” International Immunopharmacology 139 (2024): 112807, 10.1016/j.intimp.2024.112807. 39068757

[64] X. Deng , X. Li , X. Guo , et al., “Myeloid‐Derived Suppressor Cells Promote Tumor Growth and Sorafenib Resistance by Inducing FGF1 Upregulation and Fibrosis,” Neoplasia 28 (2022): 100788, 10.1016/j.neo.2022.100788. 35378464 PMC8980488

[65] F. Chen , Y. Fang , X. Chen , R. Deng , Y. Zhang , and J. Shao , “Recent Advances of Sorafenib Nanoformulations for Cancer Therapy: Smart Nanosystem and Combination Therapy,” Asian Journal of Pharmaceutical Sciences 16, no. 3 (2021): 318–336, 10.1016/j.ajps.2020.07.003. 34276821 PMC8261086

[66] L. Gopakumar , M. Sreeranganathan , S. Chappan , et al., “Enhanced Oral Bioavailability and Antitumor Therapeutic Efficacy of Sorafenib Administered in Core‐Shell Protein Nanoparticle,” Drug Delivery and Translational Research 12, no. 11 (2022): 2824–2837, 10.1007/s13346-022-01142-5. 35678961

[67] J. Wang , R. Liu , Y. Zhao , et al., “Novel Microcrystal Formulations of Sorafenib Facilitate a Long‐Acting Antitumor Effect and Relieve Treatment Side Effects as Observed With Fundus Microcirculation Imaging,” Frontiers in Oncology 11 (2021): 743055, 10.3389/fonc.2021.743055. 34513717 PMC8426437

[68] X. Yao , C. R. Zhao , H. Yin , K. Wang , and J. J. Gao , “Synergistic Antitumor Activity of Sorafenib and Artesunate in Hepatocellular Carcinoma Cells,” Acta Pharmacologica Sinica 41, no. 12 (2020): 1609–1620, 10.1038/s41401-020-0395-5. 32300243 PMC7921114

[69] H. Li , Y. Yu , Y. Liu , et al., “Ursolic Acid Enhances the Antitumor Effects of Sorafenib Associated With Mcl‐1‐Related Apoptosis and SLC7A11‐Dependent Ferroptosis in Human Cancer,” Pharmacological Research 182 (2022): 106306, 10.1016/j.phrs.2022.106306. 35714823

[70] S. Man , J. Yao , P. Lv , Y. Liu , L. Yang , and L. Ma , “Curcumin‐Enhanced Antitumor Effects of Sorafenib via Regulating the Metabolism and Tumor Microenvironment,” Food & Function 11, no. 7 (2020): 6422–6432, 10.1039/c9fo01901d. 32613952

[71] D. Singh , M. A. Khan , D. Mishra , et al., “Apigenin Enhances Sorafenib Anti‐Tumour Efficacy in Hepatocellular Carcinoma,” Translational Oncology 43 (2024): 101920, 10.1016/j.tranon.2024.101920. 38394865 PMC10899070

[72] T. Sun , W. Mao , H. Peng , Q. Wang , and L. Jiao , “YAP Promotes Sorafenib Resistance in Hepatocellular Carcinoma by Upregulating Survivin,” Cellular Oncology (Dordrecht) 44, no. 3 (2021): 689–699, 10.1007/s13402-021-00595-z. PMC12980756 33655469

[73] Q. Zhu , H. Ren , X. Li , et al., “Silencing KIF14 Reverses Acquired Resistance to Sorafenib in Hepatocellular Carcinoma,” Aging (Albany NY) 12, no. 22 (2020): 22975–23003, 10.18632/aging.104028. 33203790 PMC7746348

[74] S. Xu , S. Ling , Q. Shan , et al., “Self‐Activated Cascade‐Responsive Sorafenib and USP22 shRNA co‐Delivery System for Synergetic Hepatocellular Carcinoma Therapy,” Advanced Science 8, no. 5 (2021): 2003042, 10.1002/advs.202003042. 33717848 PMC7927615

[75] Q. Liu , N. You , J. Li , et al., “Camrelizumab Plus Sorafenib Versus Sorafenib Monotherapy for Advanced Hepatocellular Carcinoma: A Retrospective Analysis,” Frontiers in Oncology 11 (2021): 694409, 10.3389/fonc.2021.694409. 34737945 PMC8560727

